# Simultaneous quantification of bone erosions and enthesiophytes in the joints of patients with psoriasis or psoriatic arthritis - effects of age and disease duration

**DOI:** 10.1186/s13075-018-1691-z

**Published:** 2018-08-31

**Authors:** David Simon, Arnd Kleyer, Francesca Faustini, Matthias Englbrecht, Judith Haschka, Andreas Berlin, Sebastian Kraus, Axel J. Hueber, Roland Kocijan, Michael Sticherling, Juergen Rech, Georg Schett

**Affiliations:** 1 Friedrich-Alexander-University Erlangen-Nürnberg (FAU), Department of Internal Medicine 3 – Rheumatology and Immunology, Universitätsklinikum Erlangen, Ulmenweg 18, D-91054 Erlangen, Germany; 2 St. Vincent Hospital, Medical Department II, the VINFORCE Study Group, Academic Teaching Hospital of Medical University of Vienna, Vienna, Austria; 3 Department of Dermatology, University of Erlangen-Nuremberg, Erlangen, Germany

**Keywords:** Psoriatic arthritis, Psoriasis, Bone erosions, Enthesiophytes, Computed tomography, Physical function

## Abstract

**Background:**

Comprehensive simultaneous quantification of bone erosion and enthesiophytes in the joints of patients with psoriatic arthritis (PsA) has not been performed. Herein, we aimed to compare the extent of bone erosion and enthesiophytes in patients with PsA, psoriasis (PSO) and healthy controls, assess the influence of age and disease duration on the development of erosions and enthesiophytes and define their impact on physical function.

**Methods:**

Patients with PsA or with PSO and controls were analysed by high-resolution peripheral quantitative computed tomography (HR-pQCT). The extent of bone erosions and enthesiophytes was assessed and plotted according to different categories of age, duration of PSO and duration of PsA, respectively. In addition, demographic and disease-specific data, including physical function (health assessment questionnaire) were collected.

**Results:**

A total of 203 patients were analysed; 101 had PsA, 55 had PSO and 47 were healthy individuals. Patients with PsA had significantly more and larger erosions (*p* = 0.002/*p* = 0.003) and enthesiophytes (*p* < 0.001) compared to patients with PSO and healthy controls. Patients with PSO and healthy controls did not differ in erosions, while enthesiophytes were more frequent in patients with PSO than in healthy controls. Bone erosions, but not enthesiophytes, showed strong age-dependency in all three groups. In contrast, enthesiophytes were mostly influenced by the duration of PSO and PsA and, in contrast to bone erosions, were associated with poorer physical function.

**Conclusions:**

Bone erosions are age-dependent, enhanced in PsA and increase with disease duration. Enthesiophytes are less age-dependent, are enhanced in both PSO and PsA and strongly influenced by disease duration. Enthesiophytes impact physical function in PsA suggesting the need for early therapeutic interventions to prevent damage.

## Background

Psoriasis (PSO) and psoriatic arthritis (PsA) are common inflammatory disorders, which affect about 3% and 1% of the general population, respectively [1, 2]. Next to inflammation, bone destruction is a hallmark of the disease, especially in PsA. Surprisingly few studies, however, have yet comprehensively addressed bone changes in PsA. It is commonly considered that PsA is not only characterised by catabolic but also by anabolic bone pathologies, which lead to distinct architectural changes of the joints in PsA.

The development of bone erosion in the context of PsA was recognised by Gladman and colleagues 30 years ago, showing that about two thirds of patients with PsA develop radiographically evident erosions (radiographic erosions) and that their extent is related to decline in joint function [3]. Later radiographic studies showed that bone erosion start early in the course of PsA [4–7] and that greater inflammatory activity precipitates more severe erosive damage [8–10]. From the pathophysiological point of view, bone erosions in PsA result from the accumulation of osteoclasts in the joints, which is precipitated by proinflammatory cytokines [11].

Anabolic bone changes in PsA are based on new bone formation. These changes typically occur at insertion sites of tendons to bone. Enthesial inflammation is a key process in PsA and most likely triggered by mechanical stress [12–14]. Skeletal responses at the inflamed enthesial sites then result in the formation of bony spurs, also known as enthesiophytes. As compared to bone erosions, few studies have so far addressed enthesiophytes in PsA. A high-resolution peripheral computed tomography (HR-pQCT)-based study showed that enthesiophytes are key in PsA but are not found as commonly in rheumatoid arthritis [15]. Furthermore, a more recent study revealed that enthesiophytes occur early in patients with PSO without joint involvement, suggesting that they potentially reflect a common process in PSO and PSA [16].

To date, however, no comprehensive analysis of catabolic and anabolic bone damage in patients with PsA has been performed. To better interpret the impact of PsA on catabolic and anabolic bone changes, it is advantageous to investigate appropriate control populations such as healthy individuals and patients with PSO. We therefore simultaneously analysed the extent of catabolic and anabolic bone changes in healthy controls, patients with PSO and patients with PsA, by HR-pQCT and addressed the impact of age, duration of PSO and duration of PsA on respective bone changes.

## Methods

### Patients

Healthy controls and patients with PsA were part of the Erlangen Imaging Cohort (ERIC), which prospectively assesses articular bone composition in healthy controls and patients with arthritis [17]. Three groups were analysed in a cross-sectional setting: (1) patients with PsA, (2) patients with PSO and (3) healthy controls. Patients with PsA were recruited at the Rheumatology Department of the University of Erlangen-Nuremberg. All patients with PsA had to fulfil the Classification criteria for psoriatic arthritis (CASPAR) criteria [18]. An experienced rheumatologist examined the patients for clinical signs of musculoskeletal involvement (synovitis, enthesitis, dactylitis and/or inflammatory back pain) at the time of imaging. Patients with PSO were recruited at the Dermatology Department of the University of Erlangen-Nuremberg. They were referred to the Rheumatology Department of the same institution for detailed rheumatologic analysis. As with PsA, an experienced rheumatologist examined the patients for the presence of clinical signs of musculoskeletal involvement. Patients with no current or previous evidence of arthritis, enthesitis or other manifestations of PsA were selected to undergo further evaluation. In addition, healthy controls with comparable age and sex were also investigated. Healthy controls were collected by a field campaign and had no joint pain, swelling or any other sign of inflammatory disease and no personal or family history of such disease. Healthy controls had not to have osteoporosis or any evidence of metabolic diseases such as diabetes mellitus, malabsorption or thyroid dysfunction. Also, renal and hepatic function had to be normal. Subjects receiving glucocorticoids or bisphosphonates (in the present or past) were also excluded.

The study was conducted upon approval of the local ethic committee of the University of Erlangen and with the authorisation of the National Radiation Safety Agency (Bundesamt für Strahlenschutz). Each patient provided informed consent.

### Demographic and disease-specific data

Demographic data such as age, sex, body mass index (BMI) and smoking status were collected. As disease-specific parameters, disease severity of psoriasis according to the Psoriasis area severity index (PASI) [19], disease duration of PSO and PsA, involvement of nail and scalp and Health assessment questionnaire (HAQ) data were recorded. In patients with PsA the disease activity 28 score based on erythrocyte sedimentation rate (DAS28-ESR) was collected. C-reactive protein (milligrams per litre) was measured presence of rheumatoid factor and anti-cyclic citrullinated peptides antibodies (ACPA) was assessed. In addition, current medication including disease-modifying anti-rheumatic drugs (DMARDs), glucocorticoids and non-steroidal anti-inflammatory drugs (NSAIDs) was recorded.

### High-resolution peripheral quantitative computed tomography (HR-pQCT)

All subjects underwent HR-pQCT scans of the dominant hand using the XtremeCT scanner (SCANCO Medical, Brütisellen, Switzerland). To reduce movement artefacts the hand was immobilised using a custom holder. The region of interest (ROI) was the metacarpal head and the phalangeal base of the metacarpophalangeal (MCP) joints 2 and 3. During the 8.4-min scanning time the MCP region was 80 slices distal to the edge of the third metacarpal head and 242 slices proximal to it were scanned. Resolution was 82 μm isotropic voxels.

### Detection of erosions and enthesiophytes on HR-pQCT

**Fig. 1:**
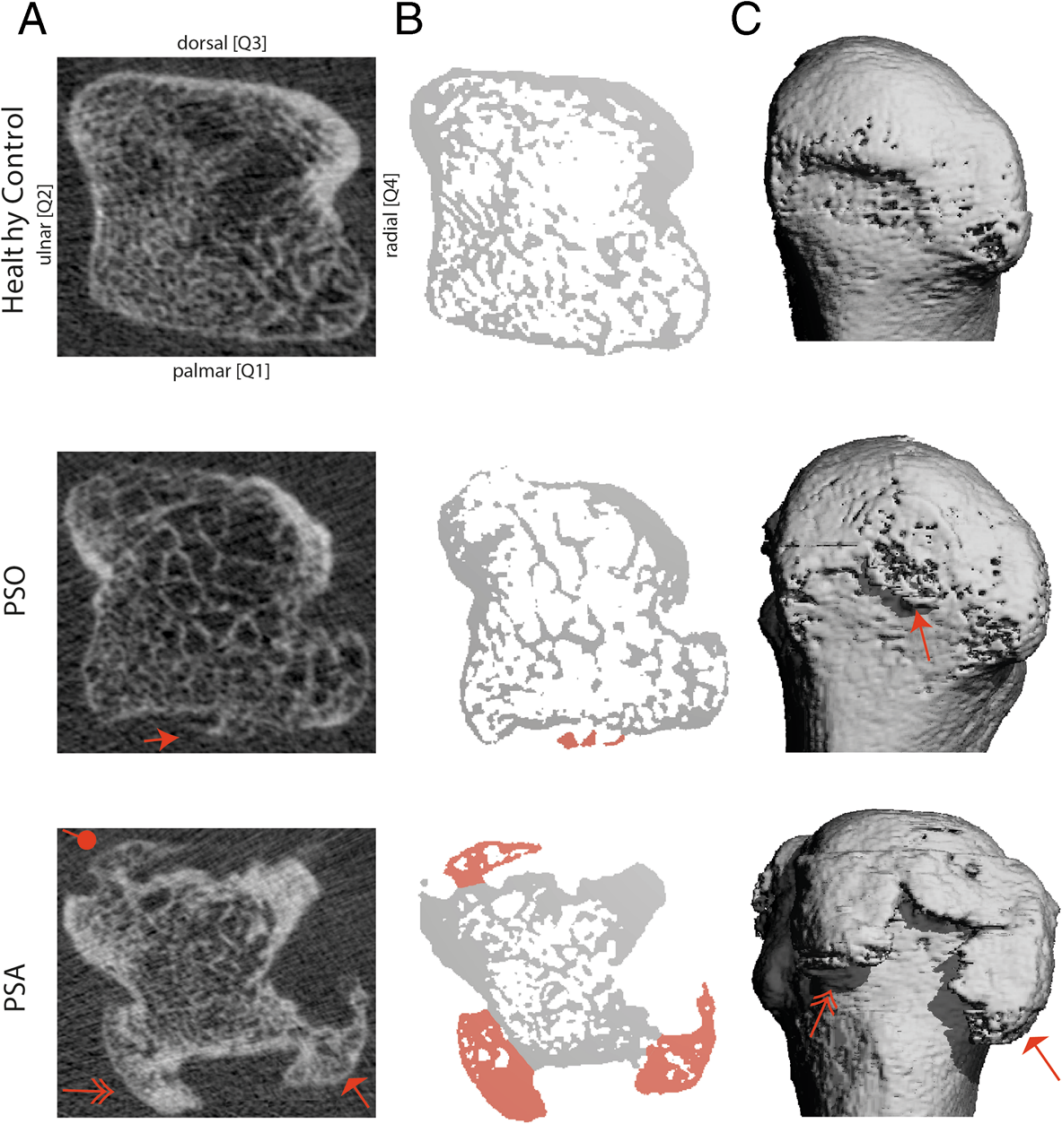
Examples of bone microstructure in healthy controls, patients with psoriasis (PSO) and patients with psoriatic arthritis (PsA). **a** Axial slices of metacarpal heads. **b** Bi-facially retouched areas of axial slices. **c** Palmar reconstruction of the associated metacarpal head. Arrows mark bone proliferation

Images were analysed using axial slices (*N* = 322). As described before [18], bone was divided into four quadrants representing the palmar (Q1), ulnar (Q2), dorsal (Q3), and radial (Q4) side, respectively. Thereby exact localization of each erosion or enthesiophyte was possible (Fig. 1). Erosions were defined as breaks in the cortical shell visible in at least two planes [20]. Enthesiophytes were identified as bony proliferations at specific anatomical sites (outside or along the insertion of the capsule). Based on previous studies we have exactly defined these anatomical sites by regions of interest (ROIs) [15–17]. If an erosion or enthesiophyte was found in one (axial) plane, the lesion had to be confirmed in the perpendicular plane (coronal or sagittal).

### HR-pQCT scoring

For scoring the number of bone erosions and enthesiophytes per patient, the sum of the respective lesion number in the four aforementioned quadrants in the four assessed regions (metacarpal head 2, metacarpal head 3, phalangeal base 2, phalangeal base 3) was calculated. If in a single quadrant more than one lesion was found, only the largest lesion was scored for its dimension. Enthesiophyte size (millimetres) was defined as the distance between the highest surface of the bone proliferation and the original surface of the cortical bone. Volume of erosions (cubic millimetres) was measured according to a half-ellipsoid formula. Images were analysed and measured by two independent and blinded readers (DS, SK) using the open source DICOM viewer Osirix V4.1 (Rosslyn, VA, USA): 3D-image software provided by the manufacturer was used for obtaining illustrative 2D and 3D images.

### Statistical analysis

Data were collected, organised and analysed through SPSS software for statistics (IBM SPSS 21.0, IBM corporation®, Armonk, NY, USA). Overall, categorical variables are presented as numbers and percentages and continuous variables are provided as mean ± standard deviation (SD) if not stated otherwise. Assumptions of normally distributed continuous variables were tested using quantile-quantile plots and the Kolmogorov-Smirnov and Shapiro-Wilk test. To explore differences in the frequency distributions of categorical variables, the chi-square (χ2) test was applied. We evaluated bivariate relationships between duration of psoriasis, duration of PsA, the HAQ and the occurrence of bone microstructural changes using Spearman correlation analysis. Analysis of the bone microstructure of subgroups (age, PSO duration, PsA duration) was by the Kruskal-Wallis test with subsequent Mann-Whitney U test for pairwise group comparisons in the case of significant Kruskal-Wallis test results. In addition, linear regression was modelled, with the total number of enthesiophytes/erosions, size of enthesiophytes and volume of erosions, respectively, as the dependent variable, while age, sex, BMI, PASI and skin disease duration were entered as independent variables. *P* values < 0.05 were regarded as significant.

## Results

### Demographic and clinical features of the patients with psoriatic disease

In total, 203 individuals were analysed for catabolic and anabolic bone changes in their joints. Among these, 101 individuals (51 women/50 men) had a diagnosis of PsA, 55 (20 women/35 men) had PSO without clinical musculoskeletal involvement and 47 individuals were healthy controls (23 women/24 men). Mean age and sex distribution were comparable among the three groups. Body mass index was significantly higher in patients with PsA or PSO compared to healthy controls, representing the well-recognised association between the disease and obesity. Smoking habits were comparable among the three groups. Patients with PsA had a mean duration of 18.9 ± 14.8 years of skin disease and 6.4 ± 7.3 years of joint disease. Patients with PSO had comparable disease duration of 15.2 ± 15.4 years: 21% of patients with PsA and 51% of patients with PSO reported of nail disease, while the prevalence of scalp involvement was 29% in patients with PSO and 20% in patients with PsA. Skin and musculoskeletal disease activity in patients with PsA, as evaluated by the PASI (3.4 ± 5.5), DAS28-ESR (2.98 ± 1.48) and HAQ (0.8 ± 0.8) was low, most likely due to effective treatment of PsA. Further disease-specific characteristics of the patient groups are shown in Table 1.

**Table 1:** Demographic and disease-specific characteristics of patients with psoriatic arthritis (PsA), patients with psoriasis (PSO) and healthy controls (HC)

	PsA	PSO	HC	*P* values
Demographic characteristics				
Number of subjects	101	55	47	–
Sex (male/female)	50/51	35/20	24/23	0.220
Age (years)	50.8 ± 13.2	49.0 ± 11.4	45.7 ± 12.9	0.056
Body mass index	28.1 ± 5.7	27.9 ± 5.6	25.0 ± 4.7	0.011
Smokers, *N* (%)	28 (27.7)	16 (29.1)	11 (23.4)	0.875
Disease specific characteristics				
Duration of PSO (years)	18.9 ± 14.8	15.2 ± 15.4	–	0.071
Duration of PsA (years)	6.4 ± 7.3	–	–	–
PASI (units)	3.4 ± 5.5	6.2 ± 8.0	–	0.007
HAQ	0.8 ± 0.8	0.4 ± 0.5	–	0.003
DAS28-ESR (units)	2.98 ± 1.48	–	–	–
Phenotypic characteristics				
Nail involvement, *N* (%)	21 (20.8)	28 (50.9)	–	0.004
Scalp involvement, *N* (%)	20 (19.8)	16 (29.1)	–	0.659
Other clinical characteristics				
Positive ACPA, *N* (%)	1 (1.0)	0	–	0.452
Positive low-titre RF, *N* (%)^a^	9 (8.9)	4 (7.3)	–	0.681
C-reactive protein (mg/L)^b^	4.9 ± 6.5	3.8 ± 4.6	–	0.228
Treatment modalities				
Current csDMARDs, *N* (%)	52 (51.5)	9 (16.4)	–	< 0.001
Current bDMARDs, *N* (%)	49 (48.5)	4 (7.3)	–	< 0.001
Current Glucocorticoids, *N* (%)	19 (18.8)	0 (0)	–	0.001
Current NSAIDs, *N* (%)	31 (30.7)	6 (10.9)	–	0.005
No systemic treatment, *N* (%)	13 (12.9)	41 (74.5)	–	< 0.001

*ACPA* anti-citrullinated protein antibody, *bDMARDs* biologic disease-modifying anti-rheumatic drugs, *csDMARDs* conventional synthetic disease-modifying anti-rheumatic drugs, *DAS28-ESR* disease activity score 28 based on erythrocyte sedimentation rate, *N* number, NSAIDs non-steroidal anti-inflammatory drugs, *PASI* Psoriasis area and severity index, *HAQ* health assessment questionnaire, *RF* rheumatoid factor

^a^< 50 IE/mL

^b^Normal value < 5 mg/mL

### Inter-observer agreement for catabolic and anabolic bone changes

Inter-observer agreement for the detection of bone lesions was very high. The intraclass correlation coefficient (ICC) was 0.96 for detection of erosions and 0.95 for detection of enthesiophytes, respectively. Regarding the extent of the lesions, the ICC for measuring the size of enthesiophytes was 0.94 and for measuring the volume of erosions it was 0.90.

### Comparison of enthesiophytes in patients with psoriasis and patients with psoriatic arthritis

In the PsA group 963 enthesiophytes were identified, while in the PSO group only 306 enthesiophytes were observed. The average number of enthesiophytes per patient was significantly higher in patients with PsA. In PsA, we detected 9.5 ± 6.7 enthesiophytes per patient, while 5.6 ± 3.3 enthesiophytes per patient were detected in PSO (*p* < 0.001) (Table 2). In both groups the majority of enthesiophytes were found at the metacarpal heads and the most affected sides were the dorsal and palmar side of the metacarpal heads, where functional entheses can be found. A significant (*p* < 0.001) difference between the PsA and PSO subgroup was detected in the extent of the enthesiophytes, with an increased enthesiophyte size of 6.8 ± 5.6 mm in the PsA group and 4.0 ± 2.2 mm in the PSO group.

**Table 2:** Anabolic and catabolic bone changes in healthy controls (HC), patients with psoriasis (PSO) and patients with psoriatic arthritis (PsA)

A. Effects of age			
*N*	HC	PSO	PsA
	(18/22/7)	(11/36/8)	(20/55/26)
Number of enthesiophytes			
20–40 years	2.41 ± 1.94	5.00 ± 2.49	7.50 ± 5.65
41–60 years	3.29 ± 1.77	6.00 ± 3.50	9.09 ± 6.20
> 60 years	3.86 ± 1.22	3.71 ± 1.98	11.96 ± 7.71
Number of erosions			
20–40 years	0.12 ± 0.33	0.27 ± 0.90	1.05 ± 1.82
41–60 years	0.33 ± 0.48	0.50 ± 0.94	1.26 ± 1.87
> 60 years	1.29 ± 0.95	0.86 ± 1.07	2.00 ± 2.95
Size of enthesiophytes (mm)			
20–40 years	1.44 ± 1.10	3.37 ± 1.61	5.26 ± 5.56
41–60 years	2.24 ± 1.15	4.40 ± 2.37	6.33 ± 4.96
> 60 years	2.69 ± 0.74	3.01 ± 1.56	8.82 ± 6.70
Volume of erosions (mm^3^)			
20–40 years	0.06 ± 0.20	0.28 ± 0.91	1.92 ± 3.61
41–60 years	0.53 ± 1.08	0.99 ± 2.25	2.95 ± 5.71
> 60 years	4.84 ± 6.31	1.99 ± 3.33	5.27 ± 12.58
B. Effects of disease duration			
*N*	PSO in PSO	PSO in PsA	PSA in PsA
	(27/21/7)	(40/40/21)	(78/17/6)
Number of enthesiophytes			
0–10 years	5.50 ± 2.49	7.91 ± 3.82	8.72 ± 5.33
11–20 years	4.80 ± 2.14	10.72 ± 7.88	10.13 ± 8.36
> 20 years	6.60 ± 4.56	11.00 ± 8.26	18.17 ± 10.93
Number of erosions			
0–10 years	0.67 ± 1.11	0.71 ± 1.45	1.31 ± 2.17
11–20 years	0.53 ± 0.92	1.33 ± 2.17	1.06 ± 1.81
> 20 years	0.20 ± 0.45	2.65 ± 2.82	3.50 ± 2.67
Size of enthesiophytes (mm)			
0–10 years	4.07 ± 1.67	5.33 ± 3.20	6.05 ± 4.59
11–20 years	3.21 ± 1.49	7.65 ± 6.60	7.61 ± 6.96
> 20 years	5.09 ± 3.19	8.38 ± 7.24	13.59 ± 9.71
Volume of erosions (mm^3^)			
0–10 years	1.21 ± 2.61	1.57 ± 3.16	2.71 ± 5.01
11–20 years	1.03 ± 2.29	3.08 ± 9.75	4.87 ± 14.36
> 20 years	0.18 ± 0.39	6.72 ± 9.24	7.44 ± 11.41

*N* numbers of subjects in the three different age categories

### Comparison of erosions in patients with psoriasis and patients with psoriatic arthritis

In the PsA group 140 erosions were detected, while in the PSO group 27 erosions were detected. With respect to the number of erosions there was a significant difference between the PsA and PSO subgroup (*p* = 0.002) with patients with PsA having more erosions (1.4 ± 2.2) than patients with PSO (0.5 ± 0.9). The sides most affected by erosions were the radial sides of the metacarpal heads. There was a significant difference in the volume of erosions between patients with PsA and patients with PSO (*p* = 0.003), with higher erosion volumes in PsA (6.3 ± 9.7 mm^3^) than in PSO (3.3 ± 3.1 mm^3^).

### Comparison of erosions and enthesiophytes in patients with psoriasis or psoriatic arthritis and healthy controls

We then compared catabolic and anabolic bone damage in patients with PsA with the ones identified in healthy controls. The number (*p* = 0.002) and size (*p* = 0.004) of bone erosions was significantly greater in patients with PsA compared to healthy controls. Furthermore, analysis of enthesiophytes showed a similar picture: again the number and size of enthesiophytes (both *p* < 0.001) was significantly greater in patients with PsA than in healthy controls. As already described [16], patients with PSO did not differ in the number of erosions compared to healthy controls, but in PSO the number of enthesiophytes was significantly greater (*p* < 0.001).

### Influence of age on bone erosions in psoriasis and psoriatic arthritis

**Fig. 2:**
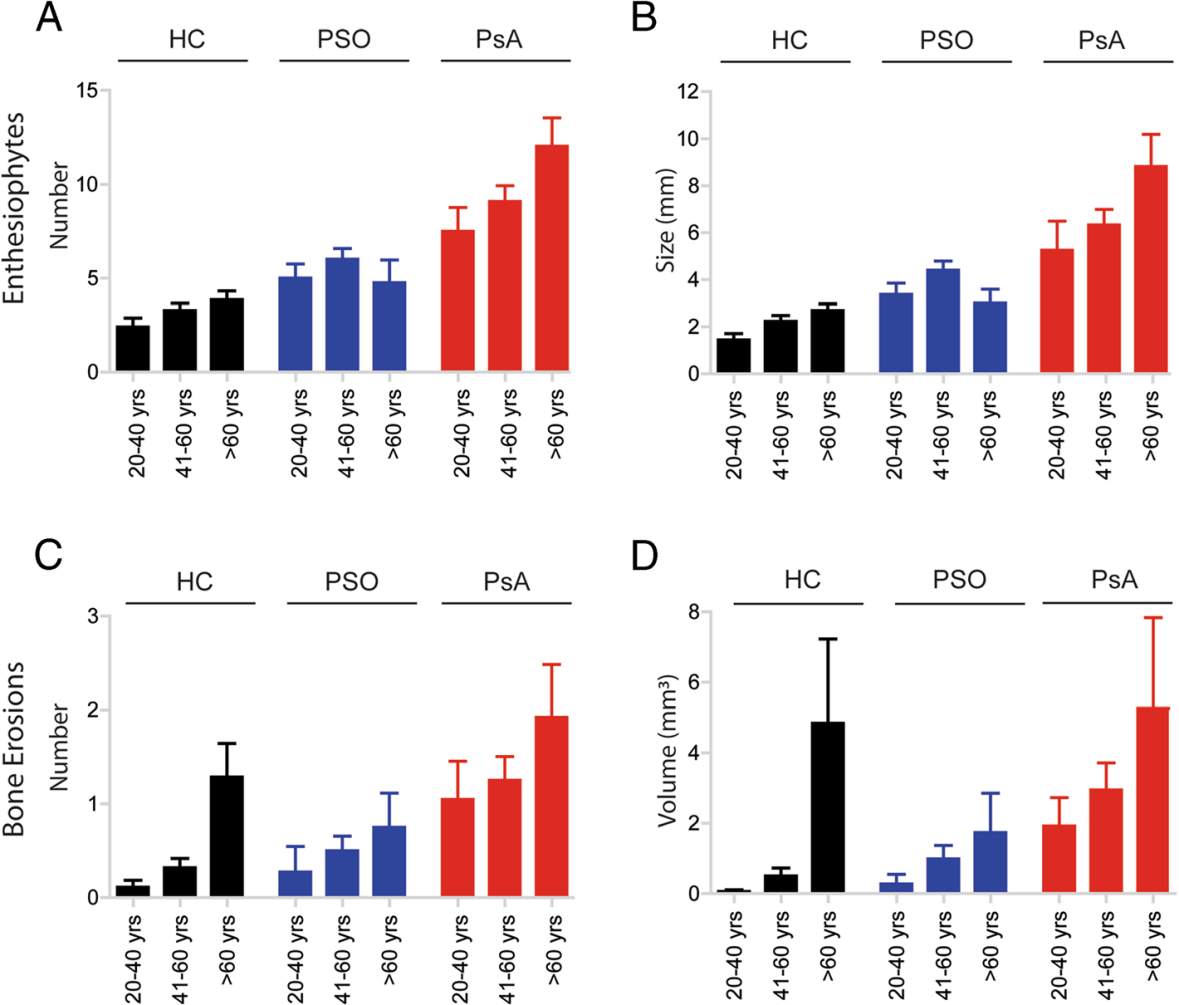
Effect of age on anabolic and catabolic bone changes in healthy controls (HC), patients with psoriasis (PSO) and psoriatic patients with arthritis (PsA). Mean (SE) values of enthesiophyte number (**a**), enthesiophyte size (**b**), erosion number (**c**) and erosion volume (**d**) in healthy controls (black), patients with PSO (blue) and patients with PsA (red) in the different age categories: (i) 20–40 years, (ii) 41–60 years and (iii) over 60 years

The analysis of number and extent of bone erosions in three age groups (20–40 years, 41–60 years and more than 60 years) revealed a continuous increase in erosive bone changes with age in healthy controls and in patients with PSO or PsA (Table 2, Fig. 2), suggesting that age is a key determinant of erosive damage in the joints. Nonetheless, patients with PsA were most affected by erosive bone damage among all age groups. Hence, the amount of erosive bone changes in a 20 to 40-year old patient with PsA was as high as in healthy individuals aged over 60 years. Healthy individuals and patients with PSO did not substantially differ in erosive changes. While erosions were absent in young healthy individuals and patients with PSO, they significantly increased with age over 60 years.

### Influence of age on enthesiophytes in psoriasis and psoriatic arthritis

Age did not influence the burden of enthesiophytes in healthy controls and patients with PSO. In patients with PsA, the number of enthesiophytes moderately increased with age (20–40 years, 7.50 ± 5.65; 41–60 years, 9.09 ± 6.20, *p* = 0.015; > 60 years, 11.96 ± 7.71, *p* = 0.001). Similarly, the size of enthesiophytes increased with age in patients with PsA (20–40 years, 5.26 ± 5.56 mm; 41–60 years, 6.33 ± 4.96 mm, *p* = 0.006; 20–40 years, 5.26 ± 5.56 mm; > 60 years, 8.82 ± 6.70 mm, *p* = 0.001).

### Influence of disease duration on bone erosions in psoriasis and psoriatic arthritis

**Fig. 3:**
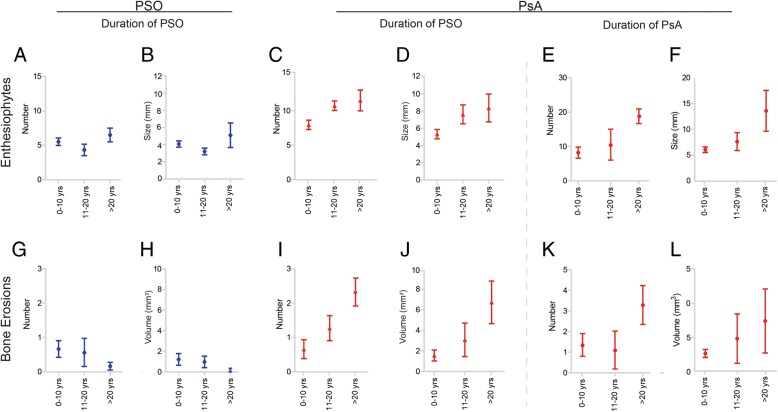
Effect of disease duration on anabolic and catabolic bone changes in patients with psoriasis (PSO) and patients with psoriatic arthritis (PsA). **a**-**d, g**-**j** Disease duration of PSO: mean (SE) values for number (**a**, **c**) and size (**b**, **d**) of enthesiophytes and number (**g**, **i**) and size (**h**, **j**) of bone erosions in patients with PSO (blue) and patients with PsA (red) in three subcategories according to duration of PSO (0–10 years, 11–20 years, more than 20 years). **e**, **f**, **k**, **l** Disease duration of PsA: mean (SE) values for number (**e**) and size (**f**) of enthesiophytes and number (**k**) and volume (**l**) of bone erosions in patients with PsA in three subcategories according to duration of PsA (0–10 years, 11–20 years, more than 20 years)

We next analysed to which extent the duration of PSO and PsA influence the burden of erosive damage in PsA. Bone erosions significantly increased with the duration of psoriatic skin disease (Fig. 3, Table 2). Hence, their numbers (2.65 ± 2.82) and volumes (6.72 ± 9.24 mm^3^) were significantly greater in patients with PsA with psoriasis of more than 20 years duration compared those with 11–20 years (*p* = 0.015 and *p* = 0.003, respectively) and less than 10 years disease duration (both *p* < 0.001). Not surprisingly, the duration of PsA also affected erosive damage (Fig. 3, Table 2): Patients with long disease duration (> 20 years) had significantly more erosions (3.50 ± 2.67), compared to those with disease duration between 11 and 20 years (*p* = 0.019) or less than 10 years (*p* = 0.015). In patients with PSO, overall erosive changes were very mild and not related to duration of psoriasis.

### Influence of disease duration on enthesiophytes in psoriasis and psoriatic arthritis

With respect to anabolic bone changes, we found that duration of skin disease influenced enthesiophyte burden both in patients with PSO and patients with PsA. While patients with PSO had mild increases in enthesiophytes with duration of psoriasis, this effect was more pronounced in PsA (Table 2). Furthermore, duration of PsA influenced the burden of enthesiophytes as well. Number (18.17 ± 10.93) and size (13.59 ± 9.71) of enthesiophytes were more than twofold greater in patients with PsA with disease duration of more than 20 years compared to those with shorter disease duration (11–20 years, *p* = 0.048; < 10 years, *p* = 0.009) (Table 2).

### Association between specific clinical variables and catabolic and anabolic microstructural changes

Duration of PSO significantly correlated with the number of enthesiophytes (*p* = 0.005), the size of enthesiophytes (*p* = 0.011), the number of erosions (*p* = 0.002) and the volume of erosions (*p* = 0.010). Duration of PsA significantly correlated with the number and size of enthesiophytes (both *p* < 0.001) and the number and volume of erosions (both *p* < 0.001). Most importantly the number and size of enthesiophytes but not number and volume of erosions correlated with physical function as measured by the HAQ score (*p* = 0.001, *p* = 0.002). Furthermore, the burden of erosions and enthesiophytes was higher (*p* = 0.028 and *p* < 0.001, respectively) in patients taking biological DMARDs compared to patients not taking biological DMARDs, reflecting the more severe disease course in patients starting biological DMARDs.

### Testing the influence of certain demographic and clinical parameters on the bone microstructure

Using a regression model including sex, age, PASI, BMI and duration of skin disease to determine the factors that independently influence erosion and enthesiophytes numbers, respectively, the only variable that remained significant was the duration of skin disease (*p* = 0.031; *p* = 0.023). Using the same model and determining the extent of erosions and enthesiophytes underlined once more the importance of the duration of skin disease, as it was the only variable influencing the size of enthesiophytes and the volume of bone erosions (*p* = 0.005 *p* = 0.034).

## Discussion

This cross-sectional study simultaneously analysed erosions and enthesiophytes in patients with PsA and compared data with findings obtained in two control populations - healthy controls and patients with PSO. The analyses revealed significantly increased catabolic and anabolic bone changes in the hand joints of patients with PsA, not only confirming the bone-destructive nature of the disease but also the co-occurrence of enthesiophytes as a typical feature of PsA. While it has already been described that erosive damage accumulates with longer disease duration, no data on enthesiophytes were available that showed their relationship with disease duration.

HR-pQCT is a highly sensitive technique for picking up bone erosions and enthesiophytes in the peripheral joints [15, 16, 20]. On the other hand, previous HR-pQCT studies have shown that healthy individuals also have a certain degree of intra-articular bone changes [16, 20]. Therefore, bone damage observed in PsA has to be validated against data obtained in healthy individuals. Furthermore, recent data has also revealed specific bone changes in PSO in the absence of clinical joint disease, particularly enthesiophytes [16]. To consider these findings we also included a group of patients with psoriasis in this analysis. Compared to healthy controls and patients with PSO the burden of structural damage was significantly higher in PsA, showing more bone erosions and more enthesiophytes.

Bone erosions were dependent on age and disease duration of PsA. Erosions reflect accumulating mechanical and inflammatory damage in the joints. In accordance, erosive damage was greatest in patients with PsA. No difference was found between healthy controls and patients with PSO. A remarkable finding was that with increase in age, bone erosions did not only increase in patients with PsA, but also in patients with PSO and healthy controls. While individuals without inflammatory joint disease (healthy controls and patients with psoriasis) most likely accrue damage due to mechanical triggers, in patients with PsA the additional inflammatory trigger appears to speed up erosions. Hence, the average 20 to 40-year old patient with PsA already exhibits a burden of bone erosions, which equals that of an individual who is over 60 years old and does not have inflammatory joint disease. These findings also indicate that judgement on the burden of erosive bone damage in an individual with PsA has to consider age as an important influencing factor. The influence of inflammation as an enhancer of progression of bone erosions is also supported by the notion that the duration of PsA is associated with the burden of bone erosions. This clinical observation is substantiated by earlier mechanistic data showing that key cytokines involved in the pathogenesis of psoriatic disease, like IL-17 and TNF-alpha, potently affect bone homeostasis by enhancing bone resorption while suppressing bone formation [21–24]. Hence the longer the bone is exposed to the inflammatory micro-environment in PsA, the more likely bone erosion occurs.

In contrast to bone erosions, enthesiophytes were not only increased in PsA but also in PSO. This finding supported previous findings showing that enthesiophytes are also present in PSO [16]. Furthermore, age dependency of enthesiophytes is less pronounced. These anabolic bone changes appear to be specifically induced by the disease itself, highlighting the role of enthesial inflammation in PsA and providing an opportunity to detect musculoskeletal involvement very early in the process of psoriatic disease. Longitudinal studies will be needed to clarify whether such lesions predict the onset of PsA. Enthesiophytes are formed at sites of functional entheses substantiating the concept of the “synovio-entheseal complex”, which is based on biomechanical stress- induced inflammation [12–14]. Given that enthesiophytes likely represent irreversible lesions, these findings particularly stress the importance of early therapeutic interventions in PsA. This consideration is even more important since our analyses showed that primarily enthesiophytes, but not bone erosions, impact the physical function of Patients with PsA.

Our data show that PsA, at present, is still associated with significant bone destructive changes, most likely because of late recognition of the disease and consequently late start of anti-inflammatory treatment [25]. These findings thereby foster the importance of implementation of early and tightly controlled treatment regimen in PsA in order to prevent the development of bone damage. In this context it is noteworthy that several different biological DMARD treatments have already shown structural benefits in the treatment of PsA, hence providing the possibility to interfere at least with the catabolic aspect of bone damage in PsA [26–28].

## Conclusions

In summary, PsA acts as a strong enhancer of age-related catabolic bone damage. In contrast, enthesiophytes, as signs of anabolic bone damage, are less age-dependent but primarily depend on the duration of PsA. Small enthesiophytes occur before clinical joint involvement and increase in size with progressive disease. Taken together, these findings highlight the destructive nature of PsA and the necessity for an early intervention to limit the burden of bone damage in PsA.

## Abbreviations

ACPA: Anti-cyclic citrullinated peptides antibodies
BMI: Body mass index
CASPAR: Classification criteria for psoriatic arthritis
DAS28-ESR: Disease activity 28 score based on erythrocyte sedimentation rate
DMARDs: Disease-modifying anti-rheumatic drugs
HAQ: Health assessment questionnaire
HR-pQCT: High-resolution peripheral quantitative computed tomography
IL-17: Interleukin-17
MCP: Metacarpophalangeal
NSAIDs: Non-steroidal anti-inflammatory drugs
PASI: Psoriasis Area Severity Index
PsA: Psoriatic arthritis
PSO: Psoriasis
ROI: Region of interest
TNF: Tumour necrosis factor
